# The influence of early diet quality on the mental health of college students: the mediation effects of height and *qi*-deficiency

**DOI:** 10.3389/fpubh.2024.1363866

**Published:** 2024-04-09

**Authors:** Xinzhu Wang, Xinyu He, Kaixian Fu, Yuxia Zhang

**Affiliations:** Xichang College, Xichang, China

**Keywords:** early diet quality, mental health, height, *qi*-deficiency, Chinese traditional medicine

## Abstract

**Background:**

In China, the prevalence of mental health issues among college students is a significant concern in society. This study aims to investigate the impact of early dietary quality on the psychological well-being of college students and elucidate the underlying mechanisms through which these effects occur, specifically focusing on height and *qi*-deficiency as mediators according to Chinese traditional medicine (CTM).

**Methods:**

A total of 655 college students were surveyed in October 2023 using paper-pencil-based questionnaires at four second-tier universities in Sichuan Province. The assessment included mental health, height, and *qi*-deficiency. Pearson’s correlation and linear regression analyses were employed to examine the mediation model and test the hypotheses.

**Results:**

The college students exhibited acceptable levels of early diet quality (*M* = 3.72) and mental health (*M* = 3.63), while also presenting mild *qi*-deficiency symptoms (*M* = 2.25). Their average height was measured at 164.61 cm. Early diet quality demonstrated significant associations with mental health (*r* = 0.38, *p* < 0.01), height (*r* = 0.32, *p* < 0.01), and *qi*-deficiency (*r* = −0.32, *p* < 0.01). Mental health displayed correlations with height (*r* = 0.32, *p* < 0.01) and *qi*-deficiency (*r* = −0.49, *p* < 0.01). The results of linear regression analyses revealed significant associations between early diet quality and mental health (*β* = 0.31, *p* < 0.01), height (*β* = 0.21, *p* < 0.01), as well as *qi*-deficiency (*β* = −0.26, *p* < 0.01). Furthermore, when early diet quality was included in the regression model, both height (*β* = 0.21, *p* < 0.01) and *qi*-deficiency (*β* = −0.35, *p* < 0.01) emerged as significant mediators in the relationship with mental health.

**Conclusion:**

The mediation model and hypotheses were strongly supported, demonstrating that early diet quality exerted an influence on the mental health of college students through two distinct pathways: height and *qi*-deficiency. Moreover, the mediating effect of *qi*-deficiency was found to be more pronounced than that of height in the relationship between early diet quality and mental health among college students.

## 1 Introduction

In recent years, the mental health concerns of college students in China have garnered significant attention. From 2010 to 2020, there was a notable increase in the prevalence rates of anxiety, depression, sleep disturbances, and suicide attempts among this population (1). Previous research has demonstrated that college students’ mental health concerns can be partially attributed to early adversity, particularly social and psychological stressors such as abuse, emotional neglect, and familial poverty experienced during childhood (2, 3). Among early adversities, the long-term impact of early diet quality on college students’ mental health has received limited attention. Drawing upon contemporary research and Chinese traditional medicine theory (CTM), this study aims to elucidate the underlying mechanism through which early diet quality exerts enduring effects on the mental well-being of college students.

Early-life poor diet quality has been found to be associated with mental health outcomes across an individual’s lifespan. A study revealed that malnutrition (including anemia and developmental delays) at the age of 3 years significantly predicts the onset of schizophrenia at the age of 23 years, with intelligence quotient (IQ) at 11 years old acting as a mediating variable (4). Children who experienced malnutrition at the age of 3 exhibited heightened levels of aggression or hyperactivity by the age of 8, demonstrated increased externalizing problems by the age of 11, and displayed more pronounced behavioral disorders and excessive exercise patterns by the age of 17, irrespective of their psychosocial adversities. A dose–response relationship was observed between the severity of malnutrition at age 3 and externalizing behavior issues at ages 8 and 17. The association between malnutrition and externalizing behavior problems is mediated by low IQ (5). A longitudinal cohort study revealed a significant correlation between early-life malnutrition and the development of paranoid, schizoid, avoidant, and dependent personality traits in individuals during middle age (6).

Evidence suggests that the relationship between early diet quality and adult mental health may be mediated by physical well-being. Firstly, the quality of one’s diet during early life is a robust predictor of physical health in adulthood. A study demonstrated that individuals who consume nutrient-rich foods (such as meat, fish, and milk) less frequently during late childhood exhibit poorer health outcomes later in life. They are more susceptible to chronic diseases, experience higher rates of acute illnesses, report increased levels of physical discomfort or pain, and display stunted growth (7). The findings of another study indicate that nutritional status during childhood (including experiences of starvation and infrequent consumption of fish and meat) can serve as a significant predictor for early physical health, specifically height as a proxy variable, as well as long-term physical health outcomes beyond the age of 30 years (8). Based on data from the China Health and Pension Follow-up Survey (2013–2015), researchers discovered a positive correlation between childhood malnutrition and an increased susceptibility to various chronic illnesses as well as diminished cognitive abilities among older individuals (9). Other similar studies have also proved that the quality of diet in childhood is closely related to the lifelong physical health of individuals (10, 11).

Furthermore, compelling evidence exists that elucidates the intricate relationship between physical and mental health. A longitudinal study encompassing a sample size of 10,693 individuals revealed that prior physical well-being can significantly impact one’s present mental well-being (12). Naylor et al. proposed that a significant proportion of clinical mental illnesses can be largely attributed to suboptimal physical health (13). Inadequate nutrition, particularly during childhood, can augment vulnerability to infectious diseases, thereby precipitating immunological dysfunction (14), and immunological dysfunction is considered as a shared and common mechanism underlying both mental and physical illness in adulthood (15). According to CTM, physical and mental health are inseparable, with physical health serving as the fundamental basis for mental well-being. Empirical evidence has demonstrated that enhancing an individual’s physical condition can yield favorable outcomes in terms of psychological disorders (16).

The state of *qi* in CTM serves as a reliable indicator of physical health status. *Qi*, one of the fundamental substances constituting the human body, is regarded as an essential form of energy and power within the body. *Qi*-deficiency refers to a lack of vitality that hinders overall bodily empowerment (17). People with evident *qi-*deficiency are more prone to physical ailments. Generally, the presence of insufficient energy and vitality, accompanied by fatigue and weariness, can be attributed to *qi*-deficiency (18). Prolonged *qi*-deficiency results in vascular blood stasis, ultimately leading to somatic discomfort and, in severe cases, atherosclerotic plaques within the vasculature, which are primary etiological factors of cardiovascular and cerebrovascular diseases (19). The inadequate generation of *qi* in the body due to poor diet and hunger leads to *qi*-deficiency, thereby adversely affecting both physical and mental well-being (17). The lifelong *qi*-deficiency constitution can be easily attributed to a prolonged inadequate diet and childhood malnutrition, which have long-term implications on health.

Multiple studies have demonstrated that height serves as a reliable indicator of physical well-being throughout an individual’s lifespan (20). For instance, a study encompassing a substantial sample size of 67,452 individuals revealed a significant association between height and overall health outcomes (21). The early-life burden of undernutrition and disease not only contributes to childhood mortality but also engenders enduring health risks for survivors, which are evidenced by stunted growth in adulthood and susceptibility to diseases in later life (22). Cámara et al. posited that adult height, serving as an indicator of childhood health, can serve as a proxy for the association between childhood health and later-life health outcomes (23). Utilizing adult height as a surrogate measure for physical well-being in adults, Wang and Niu discovered that early nutrition and the sanitary conditions of their living environment can significantly serve as predictors of individuals’ adult physical health (8). Height is widely acknowledged as a commonly used indicator for assessing early-life circumstances and physical well-being (24, 25).

Based on the aforementioned, we postulated that the mental health issues prevalent among contemporary college students can be partially ascribed to their substandard dietary quality during early life. Inadequate dietary quality in early stages leads to compromised physical well-being, subsequently augmenting the vulnerability to mental health problems during emerging adulthood. Consequently, this study endeavors to explore the pathway linking early diet quality and mental health of college students through an examination of physical well-being proxies such as height and qi-deficiency. Thus, we put forth the following three hypotheses:

*H1*: Early diet quality affects the mental health of college students;

*H2*: Height mediates the association between early diet quality and the mental health of college students;

*H3*: *Qi*-deficiency mediates the association between early diet quality and the mental health of college students.

**Figure 1:**
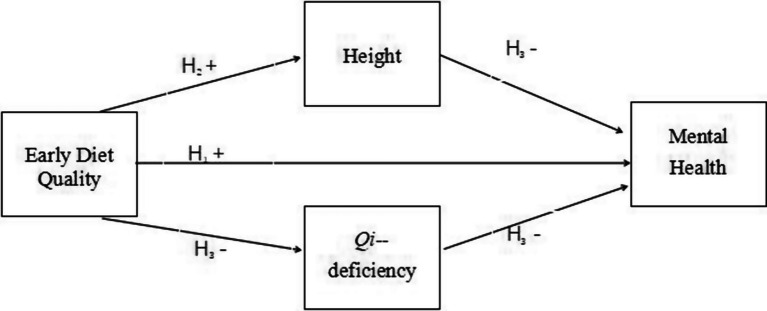
The research model and hypotheses of this study. The (+) sign denotes a positive correlation, and the (−) sign indicates a negative correlation.

The research model illustrated in Figure 1 below clearly delineates these three research hypotheses.

## 2 Research methods

### 2.1 Measurements of key variables

Mental health was measured using the WHO-5 well-being index, which has been commonly used in studies on mental health and shows good reliability and validity (26). The assessment comprises of a set of five items, wherein participants are required to provide self-reports on their mental states (e.g., moods and sleep patterns) over the preceding two-week period. In the present study, a 5-point Likert scale response was employed for this measurement, ranging from 1 (never) to 5 (all the time). One of the items states, “I have felt cheerful and been in good spirits.” The mean score of all the items was utilized to assess the level of mental health, with higher means indicating better mental well-being. In this study, the Cronbach’s alpha coefficient for the five items was 0.75, demonstrating good reliability.

The measurement of *qi*-deficiency in this study was conducted using a set of five items, which were developed based on rigorous medical research in the field of CTM (17, 27). The participants were instructed to retrospectively recall their physical sensations experienced within the past three months and provide responses to each of the five items using a 5-point Likert scale, ranging from 1 (never) to 5 (all the time). One of the items reads, “I feel fatigue after mild activities.” The Cronbach’s alpha coefficient of this scale was 0.84, indicating a high level of internal consistency and reliability. The mean score of all the items was utilized to assess the level of *qi*-deficiency, with higher means indicating declining health condition.

Previous studies have indicated that self-reported height in adults serves as a reliable proxy for their actual height (8, 28); Therefore, this study employed self-reported height as an indicator of participants’ true stature. Participants were instructed to provide their height response to the question, “how tall are you without shoes in cm?”

The early diet quality was assessed using a five-item questionnaire adapted from Cao et al.’s study (29). which asked participants to recall their dietary habits before the age of 12, including on-time consumption of breakfast, fruit intake, meat and egg consumption, and milk drinking. One of the items is: “I had breakfast on time.” The 5-point Likert scale was used with “1” indicating “never” and “5” indicating “almost every day.” The Cronbach’s alpha coefficient of 0.75 indicated good reliability in measuring early diet quality. The early diet quality index is determined by calculating the average score of the five items, with a higher score indicating better early diet quality.

### 2.2 Controlling variables

Previous research has established that the mental health of college students is linked to family residence (0 = rural area, 1 = urban area) (30), academic pressure (31), and family economic status (32). Therefore, in addition to common control variables such as sex (0 = female, 1 = male), major (0 = science, 1 = art), and age, all three aforementioned variables were controlled for in this study.

We employed a single question to assess the prevailing academic pressure, wherein participants were requested to rate their current academic pressure on a 5-point Likert scale ranging from “1” denoting “no pressure at all” to “5” indicating “very high pressure.” Simultaneously, we utilized one question to gauge the present economic status of their families, whereby participants subjectively evaluated their family’s financial condition on a 5-point Likert scale with “1” representing “very poor” and “5” signifying “very rich.”

### 2.3 Sampling procedure

We employed convenience sampling strategies to distribute 860 paper-pencil-based questionnaires among students enrolled in four second-tier universities in Sichuan Province during October 2023. Prior to conducting the survey, verbal informed consent was obtained from all participating students. Ultimately, a total of 655 valid questionnaires were collected, resulting in an effective response rate of 76.16%. The detailed characteristics of the study sample are presented in Table 1.

**Table 1:** Basic characteristics of the sample (*N* = 655).

Variables		Frequency	Percentage
Age	17–18 years old	79	12.06
	19–20 years old	190	29.01
	21–22 years old	213	32.52
	23–25 years old	173	26.41
Sex	Male	287	43.80
	Female	368	56.20
Family residence	Rural areas	403	61.53
	Urban areas	252	38.47
Major	Science	158	24.10
	Art	497	75.90

## 3 Results

### 3.1 Means, standard deviations, and correlations

Descriptive statistics and correlations are presented in Table 2.

**Table 2:** Descriptive statistics and correlation of continuous variables.

	M	SD	*EDQ*	*MH*	Height	*QD*	AP	FES	Age
*EDQ*	3.72	0.82	1						
*MH*	3.63	0.80	0.38^**^	1					
Height	164.61	8.04	0.32^**^	0.32^**^	1				
*QD*	2.25	0.74	−0.32^**^	−0.49^**^	−0.29^**^	1			
AP	3.45	1.04	−0.17^**^	−0.20^**^	−0.21^**^	0.17^**^	1		
FES	3.38	0.95	0.13^**^	0.12^**^	0.09^*^	−0.15^**^	−0.12^**^	1	
Age	20.94	1.98	0.012	−0.02	−0.02	−0.02	0.13^**^	−0.06	1

Statistically significant difference: ^*^p < 0.05, ^**^p < 0.01. The average height of the males is 170.89 cm (SD = 5.96), and the average height of the females is 159.73 cm (SD = 5.74). EDQ, Early Diet Quality; MH, Mental Health; QD, Qi-deficiency; AP, Academic Pressure; FES, Family Economic Status.

The mean scores of early diet quality and mental health were 3.72 and 3.63, respectively, slightly exceeding the midpoint value of 3.00 on the 5-point Likert scale. The average height was recorded as 164.61 cm. The average *qi*-deficiency score was found to be 2.25. Moreover, the mean academic pressure and family economic status scores were reported as 3.45 and 3.38, respectively, slightly surpassing the midpoint value of 3.00 on the 5-point Likert scale.

The results presented in Table 2 demonstrate a positive correlation between early diet quality and both mental health (*r* = 0.38, *p* < 0.01) and height (*r* = 0.32, *p* < 0.01), as well as a negative correlation with *qi*-deficiency (*r* = −0.32, *p* < 0.01). Additionally, mental health is negatively correlated with *qi*-deficiency (*r* = −0.49, *p* < 0.01) and positively correlated with height (*r* = 0.32, *p* < 0.01). Academic pressure exhibits a negative correlation with mental health (*r* = −0.20, *p* < 0.01), while family economic status shows a positive correlation with mental health (*r* = 0.12, *p* < 0.01).

### 3.2 Regression analysis

The present study employed a three-step regression technique to discern the mediating roles among the variables (33). In the initial step, a regression analysis was conducted to examine the relationship between an independent variable, X, and a dependent variable, Y. If the regression effect was found to be significant, subsequent steps were performed. In the second step, another independent variable M was included in the regression model with X as predictors. The third step involved regressing Y on both X and M simultaneously. A mediating role for M would be supported if it significantly predicted Y in this final step. This study aimed to demonstrate that height and *qi*-deficiency act as significant mediators in explaining the association between early diet quality and mental health.

In step 1 (Table 3), the regression analysis examined the association between early diet quality and mental health, while in step 2 (Table 3), separate regression analyses were conducted to explore the relationships between early diet quality and both height and *qi-*deficiency.

**Table 3:** Mediation effect analysis based on a three-step regression.

	Step 1		Step 2				Step 3	
	*MH*		Height		*QD*		*MH*	
	*β*	*t*	*β*	*t*	*β*	*t*	*β*	*t*
(Constant)		5.63^**^		60.35^**^		10.00^**^		0.063
Sex	−0.00	−0.07	−0.62	−23.77^**^	0.04	0.95	0.14	3.08^**^
FR	0.19	5.12^**^	0.12	4.69^**^	−0.12	−3.10^**^	0.12	3.55^**^
Age	0.00	0.02	−0.00	−0.17	−0.04	−0.95	−0.01	−0.33
Major	0.06	1.62	0.13	4.91^**^	−0.04	−1.12	0.02	0.51
AP	−0.12	−3.35^**^	−0.07	−2.68^**^	0.10	2.61^**^	−0.07	−2.18^*^
FES	0.05	1.32	0.02	0.86	−0.09	−2.43^*^	0.01	0.34
*EDQ*	0.31	8.57^**^	0.21	8.01^**^	−0.26	−6.88^**^	0.18	5.01^**^
Height							0.21	4.13^**^
QD							−0.35	−9.93^**^
*F*	23.29		126.06		15.12		36.93	
*Adj*.R^2^	0.19		0.57		0.13		0.33	

Statistically significant difference: ^*^p < 0.05, ^**^p < 0.01. FR, Family Residence; EDQ, Early Diet Quality; MH, Mental Health; QD, Qi-deficiency; AP, Academic pressure; FES, Family Economic Status. Coding: male = 1, female = 2; rural area = 1, urban area = 2; science = 1, art = 2.

The results of Step 1 demonstrated a significant and negative association between early diet quality and mental health (*β* = 0.31, *p* < 0.01), thus confirming the first hypothesis.

Regarding the controlling variables, we observed a significant association between family residence and mental health (*β* = 0.19, *p* < 0.01), suggesting that individuals from urban areas exhibited superior mental well-being compared to their rural counterparts. Additionally, academic pressure demonstrated a negative correlation with mental health (*β* = −0.12, *p* < 0.01). Age, sex, major, and family economic status did not yield statistically significant effects on mental health.

Step 2 revealed a significant positive association between early diet quality and height (*β* = 0.21, *p* < 0.01), as well as a negative association with *qi*-deficiency (*β* = −0.26, *p* < 0.01).

Subsequently, in step 3, we performed a multiple regression analysis to examine the simultaneous effects of height, *qi*-deficiency, and early diet quality on mental health. The detailed results can be found in Table 3.

The regression results from step 3 demonstrated that, in the presence of height and *qi*-deficiency, early diet quality continued to exert significant effects on mental health (*β* = 0.18, *p* < 0.01), albeit with a smaller effect size compared to step 1 (*β* = 0.31, *p* < 0.01).

In addition, after controlling for early diet quality in the regression analysis, it was found in step 3 that height (*β* = 0.21, *p <* 0.01) and *qi*-deficiency (*β* = −0.35, *p* < 0.01) exerted significant effects on mental health.

Therefore, height and *qi*-deficiency partially mediated the relationship between early diet quality and mental health, indicating that hypothesis 3 is well supported.

**Figure 2:**
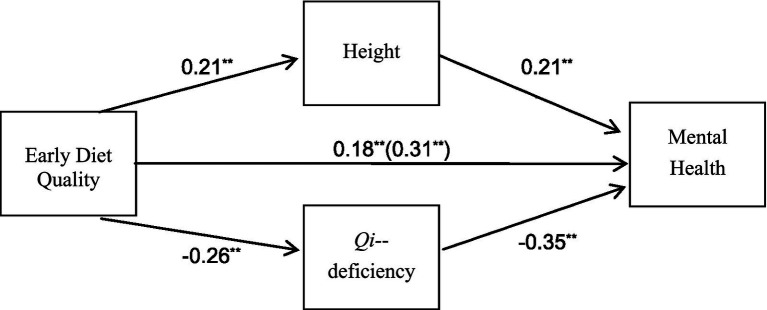
The research model and hypotheses in this study.

The mediating role of height and *qi-*deficiency is illustrated in Figure 2.

The overall impact of the pathway “early diet quality → mental health” was 0.31, with an indirect effect through the pathway “early diet quality → height → mental health” of 0.04 (i.e., 0.21 * 0.21). Therefore, the mediating role of height between early diet quality and mental health accounted for approximately 12.90% (i.e., 0.04/0.31) of the total effect. Similarly, the indirect effect through the pathway “early diet quality → *qi*-deficiency → mental health” was 0.091 (i.e., 0.26 * 0.35). Hence, the mediating effect of *qi*-deficiency between early diet quality and mental health contributed to around 29.03% (i.e., 0.09/0.31) of the total effect. The mediating effect of *qi*-deficiency exhibited a greater magnitude compared to height in the association between early diet quality and the mental health of college students.

## 4 Discussion

### 4.1 Descriptive statistics for early diet quality, mental health, height, and *qi*-deficiency

The findings of this study indicate that the average early diet quality score among college students in China exceeded the midpoint value of 3.00 on the 5-point Likert scale. It can be inferred that the early diet quality of college students was deemed acceptable rather than excellent. This conclusion aligns with previous studies conducted between 2010 and 2017, which highlighted malnutrition as an ongoing public health concern among Chinese adolescents (34, 35).

The mean mental health score slightly exceeded the midpoint value of 3.00 on the 5-point Likert scale, indicating that college students exhibited an acceptable rather than excellent level of mental health status. This finding aligns with previous studies highlighting the prevalence of mental health issues among Chinese college students (1).

In this study, the average height of college students was found to be 164.61 cm (170.89 cm for males and 159.73 cm for females), which surpassed the mean height observed among Chinese adults aged 18 to 44 in the years spanning from 2015 to 2019 (169.70 cm for males and 158.00 cm for females) (36). The primary reason for this outcome is primarily attributed to the fact that college students, on average, exhibit a greater height compared to the general adult population.

College students reported an average *qi*-deficiency score of 2.25 on the 5-point Likert scale, indicating a mild state of *qi*-deficiency among college students based on CTM principles. Since no prior surveys have been conducted on *qi*-deficiency specifically in this population, direct comparisons with other studies are not feasible.

### 4.2 Relationship between early diet quality and the mental health of college students

The first hypothesis posits that early diet quality is a significant predictor of mental health, which is strongly supported by the regression analysis (*β* = 0.31, *p* < 0.01) in step 1 (Table 3). This finding aligns with previous studies conducted by Venables and Raine (4), Liu et al. (5), and Hock et al. (6), all of which have demonstrated the substantial impact of early diet quality on mental health outcomes during adulthood.

Our second hypothesis posits that height serves as a mediator in the relationship between early diet quality and mental health, which is strongly supported by the regression analysis conducted in steps 2 and 3 (Table 3). Firstly, our findings reveal a positive predictive effect of early diet quality on height (*β* = 0.21, *p* < 0.01), indicating a significant and favorable impact of early nutrition on the physical well-being of college students. This outcome aligns with previous literature (8, 22, 37), where it has been highlighted that early nutrition and diseases are crucial non-genetic factors influencing growth and adult body height (37). Secondly, we observe a positive association between height and mental health among college students (*β* = 0.21, *p* < 0.01), suggesting that physical well-being contributes to mental well-being—a finding consistent with prior research (13, 16). For instance, Happell et al.’s literature review demonstrated the beneficial effects of improving physical status on mental health (16).

The third hypothesis, which posits that *qi*-deficiency may serve as a mediator in the relationship between early diet quality and mental health, is also strongly supported. Firstly, there was a significant predictive association between early diet quality and *qi*-deficiency (*β* = −0.261, *p* < 0.01; step 2 in Table 3). According to CTM, prolonged inadequate dietary intake or childhood illnesses are primary factors contributing to the development of a constitution characterized by *qi*-deficiency (38). For instance, suboptimal nutrition during childhood can impair the functioning of internal organs (particularly the spleen and stomach), leading to lifelong *qi*-deficiency (39). Secondly, our findings demonstrate a significant impact of *qi*-deficiency on mental health (*β* = −0.35, *p* < 0.01; step 3 in Table 3), aligning with previous research findings (40, 41). A study revealed that both sexes exhibited associations between major depression and conditions characterized by either *qi*-stagnation or *qi*-deficiency; particularly noteworthy was the link observed between major depression and male individuals with *qi*-deficiency (41).

In summary, all three hypotheses were strongly supported, indicating that early diet quality has a significant impact on the mental health of college students through the mediating pathways of height and *qi*-deficiency. Notably, the mediating effect of *qi*-deficiency was found to be greater than that of height. Given that both height and *qi-*deficiency reflect an individual’s physical health status, these findings suggest that early diet quality can indirectly influence adult mental health through its effects on physical health in adulthood.

### 4.3 Effects of controlling variables

Regarding the controlling variables, our findings indicate that individuals residing in urban areas exhibit superior mental well-being compared to their rural counterparts. This disparity can be attributed to the fact that families in urban regions of China possess greater access to family and public educational resources for their children, including financial support and educational opportunities (42). Additionally, we observed a negative association between academic pressure and mental health. This can be explained by the notion that heightened academic pressure elicits an immunological response, ultimately leading to detrimental effects on both physical and mental health (43).

### 4.4 Theoretical and practical implications

The findings of this study suggest that early dietary quality may have an indirect impact on mental health in early adulthood through its influence on physical health. This outcome aligns with the concept of health in CTM, which emphasizes the interdependence of physical and mental well-being (44). Furthermore, it is consistent with contemporary research indicating a close relationship between physical and mental illnesses, sharing common underlying mechanisms (45). For instance, previous research has demonstrated that inadequate nutrition during childhood can heighten susceptibility to infectious diseases, thereby leading to immunological dysfunction (14), which serves as a shared mechanism for both mental and physical disorders (15).

Furthermore, this study suggests that physical health acts as a mediator in the relationship between early diet quality and mental health among college students. It identifies two specific pathways, namely height and *qi*-deficiency, with the mediating effect of *qi*-deficiency being significantly stronger than that of height. Previous studies have commonly considered height as a reliable proxy for early nutrition and overall health throughout life. However, this study proposes that compared to height, *qi*-deficiency may serve as a more accurate proxy for early nutrition and long-term health outcomes. According to CTM, poor diet quality leading to *qi-*deficiency during early life can render individuals more susceptible to both physical and mental health issues across their lifespan. In summary, from a theoretical standpoint, this study elucidates the precise mechanism through which early diet quality influences mental health during young adulthood.

In practical terms, this study suggests that the prevention of psychological issues among college students should commence with their childhood experiences, including early dietary quality. The issue of dietary quality in primary schools continues to be a matter of public health concern, particularly in towns and rural areas. It is well-known that many boarding school students in these regions exhibit aversion toward the provided meals, leading to common occurrences of picky eating, breakfast skipping, and infrequent milk consumption. Evidently, such practices can have detrimental effects on their physical well-being, resulting in stunted growth and *qi*-deficiency; ultimately potentially impacting their mental health during their future college years.

Furthermore, this study can aid college students in early detection of subtle indicators of declining physical and mental well-being. In the context of CTM, *qi*-deficiency can be regarded as an initial sign of deteriorating physical and mental health. When college students experience symptoms such as excessive sweating after minimal exercise and a lack of strength, they should modify their lifestyle to restore *qi* levels in the body instead of persisting with unhealthy eating habits like consuming junk food or skipping breakfast until clinical intervention becomes necessary. To summarize, for college students who had poor dietary quality during childhood, attending to their own *qi*-deficiency status is highly beneficial for healthcare.

## 5 Strengths and shortcomings of this study

This study possesses two distinct theoretical advantages. Firstly, it highlights the significance of both height and *qi*-deficiency as mediators in the relationship between early diet quality and mental health. While previous studies have often employed height as a proxy variable for early experiences and lifelong health (21, 25), few have examined its role as an intermediary variable between early diet quality and mental health during early adulthood. Moreover, although some Chinese scholars have identified a strong correlation between *qi*-deficiency and psychological disorders such as anxiety and depression (41, 46), limited research has explored the mediating effect of *qi*-deficiency on the association between early diet quality and mental health during early adulthood. Secondly, this study demonstrates that *qi*-deficiency plays a stronger mediating role than height in linking early diet quality to mental health outcomes, suggesting that *qi*-deficiency may be a better predictor of college students’ mental well-being compared to their height. This finding aligns with the concept in CTM that emphasizes the fundamental importance of *qi*–a vital substance providing energy and power – for both physical and psychological well-being (47).

This study holds practical significance as it pertains to the prevention of mental health issues. *Qi*-deficiency serves as a valuable indicator in this regard, enabling college students to promptly adjust their lifestyle (e.g., adopting a healthier diet and prioritizing rest) upon experiencing symptoms such as excessive sweating after minimal exercise and lack of strength. While inadequate dietary habits during early life may impact height and contribute to *qi*-deficiency in adulthood, the latter can be effectively addressed through the adoption of a healthy lifestyle (17), whereas adult height remains unmodifiable. Given the Chinese government’s current emphasis on mental illness prevention, it is crucial to acknowledge the substantial influence that *qi*-deficiency exerts on mental health.

However, this study has several limitations. Firstly, we did not incorporate other physical discomforts such as stomach discomfort, menstrual pain, and cold extremities to assess the physical well-being of college students. Based on interview responses from the study participants, these symptoms are prevalent among college students. By including these indicators in our assessments, a more comprehensive measurement of physical health status can be achieved, enabling a more detailed estimation of the mediating role of height and *qi*-deficiency. Secondly, our sampling technique was non-randomized and limited to second-tier universities without representation from first-tier institutions. Consequently, the sample used in this study lacks representativeness which compromises its external validity. Future research should address these limitations to further explore the intricate relationship between early diet quality and both physical and mental health during early adulthood.

## Data availability statement

The raw data supporting the conclusions of this article will be made available by the authors, without undue reservation.

## Ethics statement

The studies involving human participants were reviewed and approved by the Research Ethics Committee of Xichang University (LG2021), and was conducted in accordance with the principles outlined in the Declaration of Helsinki. The participants provided their written informed consent to participate in this study.

## Author contributions

XW: Writing – original draft, Writing – review & editing, Methodology. XH: Conceptualization, Methodology, Writing – review & editing. KF: Data curation, Investigation, Writing – review & editing. YZ: Investigation, Validation, Writing – review & editing.
